# Long-Term Atherogenic Dyslipidaemia Burden, Rather than Visit-to-Visit Variability, Is Associated with Carotid Intima–Media Thickness

**DOI:** 10.3390/biomedicines14010226

**Published:** 2026-01-20

**Authors:** Ahmet Yılmaz, Enes Çon

**Affiliations:** Department of Cardiology, Faculty of Medicine, Karamanoğlu Mehmetbey University, Karaman 70200, Turkey; enes_con@hotmail.com

**Keywords:** triglyceride-to-HDL ratio, carotid intima media thickness, lipid variability, atherogenic dyslipidaemia, statin therapy, subclinical atherosclerosis

## Abstract

**Background/Objectives**: The triglyceride-to-High-density lipoprotein cholesterol (TG/HDL) ratio is an established marker of atherogenic dyslipidaemia and insulin resistance. Although its association with subclinical atherosclerosis has been reported, the relative contributions of long-term TG/HDL burden and visit-to-visit variability to carotid intima media thickness (CIMT) remain unclear. This study aimed to evaluate the differential associations of the longitudinal mean and temporal variability of the TG/HDL ratio with CIMT. **Methods**: This retrospective single-center observational cohort study included 260 adult patients with at least three years of longitudinal lipid measurements and a standardized carotid ultrasonography assessment. The longitudinal mean TG/HDL ratio and variability indices, including standard deviation, coefficient of variation, average real variability and variability independent of the mean, were calculated. CIMT was measured using B-mode ultrasonography. Associations were assessed using correlation analyses, multivariable linear regression, joint category analyses and stratified analyses according to statin therapy. **Results**: The longitudinal mean TG/HDL ratio was independently associated with increased CIMT after adjustment for traditional cardiovascular risk factors. In contrast, TG/HDL variability indices showed no independent association with CIMT and did not improve model performance beyond the mean TG/HDL ratio. Restricted cubic spline analysis demonstrated a significant non-linear association between TG/HDL mean and CIMT, suggesting a threshold-dependent relationship. Joint category analyses demonstrated higher CIMT values in groups with elevated TG/HDL mean regardless of variability status. A significant interaction was observed between TG/HDL variability and statin therapy (*p* for interaction = 0.011). **Conclusions**: These findings indicate that cumulative exposure to atherogenic dyslipidaemia, reflected by the long-term mean TG/HDL ratio, is more strongly associated with subclinical carotid atherosclerosis than short-term lipid fluctuations.

## 1. Introduction

Subclinical carotid atherosclerosis represents an early stage of vascular remodeling and is commonly assessed by carotid intima–media thickness (CIMT), a well-established structural marker of atherosclerotic burden. Increased CIMT has been consistently associated with aging, hypertension, diabetes mellitus, dyslipidaemia, and an elevated risk of future cardiovascular events [1]. As a non-invasive and reproducible measure, CIMT provides valuable insight into the cumulative effects of cardiometabolic risk factors on the arterial wall.

There is a growing interest in calculating atherogenic dyslipidaemia through parameters of non-traditional and composite lipids. Markers of remnant lipoproteins and lipid fractions that are typically used in isolation to measure LDL cholesterol [1,2]. Among these, the triglyceride-to-high-density lipoprotein cholesterol (TG/HDL) ratio has emerged as an integrative marker of insulin resistance, remnant lipoprotein burden, and metabolic dysfunction. Prior studies have demonstrated associations between elevated TG/HDL ratios and subclinical atherosclerosis, arterial stiffness, and adverse cardiovascular outcomes, supporting its relevance as a surrogate of atherogenic lipid exposure [3].

Concurrently, visit-to-visit lipid variability has gained interest as a potential cardiovascular risk factor independent of mean lipid levels. Large population-based studies have linked lipid variability to myocardial infarction, stroke, and all-cause mortality, suggesting that temporal fluctuations in lipid levels may reflect biological instability, treatment inconsistency, or heightened vascular vulnerability. However, whether lipid variability contributes to structural vascular remodeling, as captured by CIMT, remains uncertain. Unlike acute cardiovascular events or plaque-related phenotypes, CIMT reflects a slowly progressive process driven primarily by sustained metabolic stress rather than short-term lipid fluctuations [4,5].

From a pathophysiological perspective, atherosclerosis is increasingly recognized as a dynamic process driven by cumulative metabolic exposure over time. Mean lipid levels reflect the overall atherogenic burden to which the arterial wall is chronically exposed, whereas visit-to-visit lipid variability has been proposed to promote endothelial dysfunction through oxidative stress and inflammatory lipid cascades [6].

However, when considering structural vascular markers, CIMT primarily represents a slowly progressive remodeling process of the arterial wall. As such, CIMT is more strongly influenced by sustained lipid exposure than by short-term lipid fluctuations. This distinction suggests that lipid variability may be more relevant to dynamic plaque-related processes and acute cardiovascular events, whereas CIMT predominantly captures the cumulative effects of chronic metabolic stress. Understanding these differential roles of lipid levels and variability is essential for interpreting their respective associations with subclinical atherosclerosis and formed the rationale for the present study [7,8].

This study analyzed the relationship of the long-term mean value and the temporal fluctuation of the triglyceride-to-HDL cholesterol ratio with the carotid intima-media thickness as structural measure of subclinical atherosclerosis and each of these two dimensions of lipid factors. Most studies of the effects of lipids on the vasculature are cross-sectional or measure average lipids or averages over the study period. This study adds to the limited evidence on the effects of longitudinal lipid fluctuation on the structure of the vascular wall by considering the mean level and the temporal variability of a lipid parameter, within a single framework. This more dynamic and complete approach will provide a better understanding of the vascular risk associated with lipids.

## 2. Materials and Methods

### 2.1. Study Design and Setting

This single-center retrospective observational cohort study was conducted within the Department of Cardiology, Karamanoğlu Mehmetbey University Faculty of Medicine (Karaman Training and Research Hospital). Adult patients who qualified for routine cardiovascular follow-up and had undergone multiple assessments of their lipid profiles and carotid sonography were reviewed via their electronic medical records. Carotid sonography was performed in the cardiology clinic after longitudinal lipid follow-up due to clinical indications. The study was carried out in conformity with the Declaration of Helsinki and was approved by the Local Ethic Committee (5 March 2025, No. 17-2025/12).

### 2.2. Study Population

Eligible participants were adults aged ≥ 18 years with longitudinal lipid data comprising at least six measurements of LDL cholesterol, HDL cholesterol, and triglycerides over a minimum follow-up period of three years, as well as a carotid intima–media thickness (CIMT) assessment performed for routine clinical evaluation after completion of lipid follow-up.

Patients were excluded if they had incomplete longitudinal lipid follow-up, a history of carotid artery interventions, prior neck irradiation, or medical conditions associated with clinically significant secondary dyslipidaemia. In accordance with established recommendations for CIMT assessment, individuals with focal carotid lesions or carotid atherosclerotic plaques detected on ultrasonography were also excluded to ensure evaluation of diffuse arterial wall thickening rather than plaque-related pathology.

A total of 260 patients were included in the final analysis.

### 2.3. Data Collection and Definitions

From the datasets of the subjects concerned, we took the following information: demographics, body mass index, the presence or absence of diabetes and hypertension, smoking status, use of statins and the presence or absence of coronary artery disease. The ratio of triglyceride to high-density lipoproteins (TG/HDL) was determined from the attended visits and the longitudinal mean TG/HDL ratio (TG/HDL mean) was calculated.

TG/HDL mean for the visits was calculated and to determine visit-to-visit TG/HDL variability, standard deviation (sd), coefficient of variation (cv), average real variability (arv) and variability independent of the mean (vim) were determined from the expression: sd/mean ^β, where β was obtained from log-log regression. Descriptive purposes served the variability index calculations for individual lipid fractions. TG/HDL variability was defined as the visit-to-visit temporal fluctuation of the TG/HDL ratio across longitudinal clinical visits, reflecting temporal lipid instability over time.

### 2.4. Carotid Ultrasonography

Carotid ultrasonography for the assessment of carotid intima–media thickness (CIMT) was performed by trained cardiologist specialists using a Mindray DC-80 ultrasound system (Mindray Medical India) equipped with high-frequency linear array transducers. CIMT measurements were obtained at end-diastole from plaque-free segments of both common carotid arteries, approximately 1 cm proximal to the carotid bulb. Measurements were preferentially acquired from the far wall to improve accuracy and reproducibility, and the mean CIMT value was calculated by averaging measurements from the right and left carotid arteries. All CIMT measurements were performed manually; no automated edge-detection or software-based measurement tools were used.

CIMT assessment was performed shortly after the final lipid measurement, ensuring that the ultrasound evaluation reflected cumulative lipid exposure over the longitudinal follow-up period.

### 2.5. Statistical Analysis

Statistical analyses were performed using IBM SPSS Statistics software (version 26.0; IBM Corp., Armonk, NY, USA) and R software (version 4.3.0; R Foundation for Statistical Computing, Vienna, Austria). Continuous variables are presented as mean ± standard deviation or median with interquartile range, as appropriate, while categorical variables are expressed as frequencies and percentages.

Associations between TG/HDL-related metrics and carotid intima–media thickness were initially assessed using Pearson or Spearman correlation analyses, depending on data distribution. Multivariable linear regression models were constructed to evaluate independent associations with mean carotid intima–media thickness (CIMT mean), adjusting for age, sex, body mass index, diabetes mellitus, hypertension, smoking status, statin therapy, and coronary artery disease. To minimize multicollinearity, TG/HDL variability indices were entered into regression models separately from the TG/HDL mean.

Quartile-based analyses and joint-category analyses were performed to examine the combined effects of long-term TG/HDL burden and its temporal variability on CIMT. Effect modification by statin therapy was evaluated by including an interaction term between TG/HDL variability indices and statin use.

To explore potential non-linear associations between the longitudinal mean TG/HDL ratio and CIMT, restricted cubic spline regression models were constructed with four knots placed at prespecified percentiles (5th, 35th, 65th, and 95th percentiles) of the TG/HDL mean distribution. All spline models were adjusted for age, sex, body mass index, diabetes mellitus, hypertension, smoking status, statin therapy, and coronary artery disease.

A two-sided *p* value < 0.05 was considered statistically significant.

## 3. Results

A total of 260 patients with longitudinal lipid measurements over a three-year period were included in the final analysis. The mean age of the study population was 58.4 ± 10.7 years, and 46.5% of participants were female. The prevalence of diabetes mellitus and hypertension was 38.8% and 54.6%, respectively, while 41.9% of patients were receiving statin therapy at baseline. A history of coronary artery disease was present in 32.3% of the cohort.

The mean carotid intima–media thickness (CIMT mean) was 0.95 ± 0.12 mm. Over the longitudinal follow-up, the average TG/HDL ratio (TG/HDL mean) was 2.73 (2.38–3.10), reflecting a moderate degree of atherogenic dyslipidaemia in the overall population. The temporal variability of the TG/HDL ratio, assessed using the variability independent of the mean (TG/HDL vim), showed a mean value of 39.6 ± 13.9, indicating substantial inter-individual heterogeneity in lipid fluctuation patterns.

**Figure 1:**
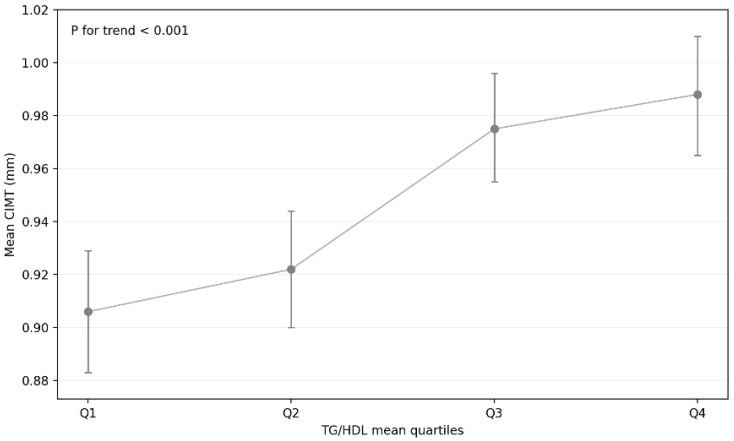
TG/HDL mean quartiles and CIMT. Error bars indicate 95% confidence intervals. A significant increasing trend in CIMT mean across TG/HDL mean quartiles was observed (*p* for trend < 0.001).

When patients were stratified according to quartiles of TG/HDL mean, those in the higher quartiles were older and exhibited a higher prevalence of diabetes mellitus and hypertension (all *p* for trend < 0.05). CIMT mean increased progressively across TG/HDL mean quartiles, suggesting a graded relationship between atherogenic dyslipidaemia burden and subclinical carotid atherosclerosis (Figure 1). In contrast, baseline demographic and clinical characteristics were largely comparable across quartiles of TG/HDL vim, with no significant differences observed in age, sex distribution, or major cardiovascular risk factors.

Baseline lipid parameters, including LDL-cholesterol, triglycerides, and HDL-cholesterol, demonstrated expected distributions, while variability indices of individual lipid fractions (LDL vim, Triglyceride vim, HDL vim, and VLDL vim) showed wide dispersion but no clear unadjusted association with CIMT mean at the descriptive level.

A detailed summary of baseline demographic, clinical, and biochemical characteristics of the study population is presented in Table 1.

**Figure 2:**
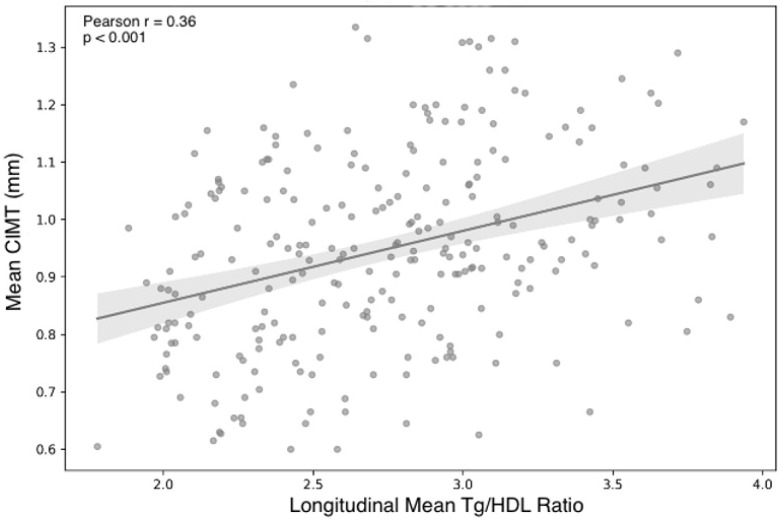
Scatter plot illustrating the association between the longitudinal mean TG/HDL ratio (TG/HDL mean) and carotid intima–media thickness (CIMT mean). Each dot represents an individual participant. The solid line indicates the fitted linear regression line, and the shaded area represents the 95% confidence interval. A significant positive association was observed between TG/HDL mean and CIMT mean (Pearson r = 0.36, *p* < 0.001).

In unadjusted analyses, the longitudinal mean TG/HDL ratio demonstrated a moderate and statistically significant positive correlation with CIMT mean. Pearson correlation analysis showed that higher TG/HDL mean values were associated with increased CIMT mean (r = 0.36, *p* < 0.001), a finding that was confirmed using Spearman rank correlation (ρ = 0.39, *p* < 0.001), indicating robustness against non-normality (Figure 2).

To further quantify this association, multivariable linear regression analyses were performed (Table 2). In the age- and sex-adjusted model (Model 1), TG/HDL mean remained significantly associated with CIMT mean (β = 0.051, 95% CI 0.026–0.076, *p* < 0.001). This relationship persisted after additional adjustment for body mass index, diabetes mellitus, hypertension, smoking status, statin therapy, and the presence of coronary artery disease (Model 2; β = 0.044, 95% CI 0.016–0.071, *p* = 0.002).

Across all models, TG/HDL mean consistently emerged as an independent determinant of CIMT, with higher atherogenic dyslipidaemia burden corresponding to greater subclinical carotid atherosclerosis (Table 2).

These findings indicate that the long-term burden of atherogenic dyslipidaemia, as reflected by the TG/HDL ratio, is independently associated with increased carotid intima–media thickness, beyond traditional cardiovascular risk factors.

To evaluate whether temporal variability in the TG/HDL ratio provides incremental information beyond its long-term mean level, TG/HDL variability indices were sequentially introduced into multivariable linear regression models (Table 3).

In unadjusted analyses, TG/HDL vim was not significantly correlated with CIMT mean (r = 0.02, *p* = 0.72). When TG/HDL vim was added to the fully adjusted model that already included TG/HDL mean and traditional cardiovascular risk factors, TG/HDL mean remained a significant independent determinant of CIMT mean (β = 0.044, 95% CI 0.016–0.071, *p* = 0.002), whereas TG/HDL vim showed no independent association with CIMT mean (β = −0.0005, 95% CI −0.0013 to 0.0003, *p* = 0.185).

Importantly, inclusion of TG/HDL vim did not materially improve model performance, as reflected by a negligible change in explained variance (ΔR^2^ < 0.002). Similar findings were observed when alternative TG/HDL variability metrics, including SD, coefficient of variation (CV), and average real variability (ARV), were examined; none demonstrated a significant association with CIMT mean after adjustment for TG/HDL mean and clinical covariates.

These results indicate that the cumulative burden of atherogenic dyslipidaemia, as captured by the longitudinal mean TG/HDL ratio, rather than short-term or visit-to-visit fluctuations, is the principal lipid-related determinant of subclinical carotid atherosclerosis in this cohort. CIMT is a chronic and slowly progressive structural process; therefore, long-term exposure (mean TG/HDL) is expected to be more decisive than short-term lipid fluctuations.

To assess the combined effect of the long-term burden of atherogenic dyslipidaemia and its temporal fluctuation, participants were categorized into four groups according to median values of TG/HDL mean and TG/HDL vim: (1) low mean/low vim (reference), (2) low mean/high vim, (3) high mean/low vim, and (4) high mean/high vim. Adjusted mean CIMT values were estimated using a general linear model controlling for age, sex, body mass index, diabetes mellitus, hypertension, smoking status, statin therapy, and coronary artery disease.

Overall, the joint grouping was significantly associated with CIMT mean (global *p* < 0.001). Compared with the reference group (low mean/low vim), CIMT mean was significantly higher in both high TG/HDL mean groups, regardless of vim status. Specifically, the high mean/low vim group exhibited the highest adjusted CIMT mean, while the high mean/high vim group also showed a significantly elevated CIMT mean. In contrast, the low mean/high vim group did not differ significantly from the reference group. These findings indicate that TG/HDL mean, rather than TG/HDL variability, primarily drives the association with CIMT when both dimensions are considered simultaneously (Table 4).

To further explore whether the association between TG/HDL variability and CIMT differed according to lipid-lowering treatment, analyses were stratified by statin therapy status (Table 5).

Among patients not receiving statin therapy, TG/HDL vim was not associated with CIMT mean after adjustment for TG/HDL mean and clinical covariates. In contrast, among statin-treated patients, higher TG/HDL vim was independently associated with lower CIMT mean, indicating a significant inverse relationship. Importantly, TG/HDL mean remained positively and independently associated with CIMT mean in both statin subgroups.

These findings suggest that statin therapy modifies the relationship between TG/HDL variability and subclinical carotid atherosclerosis, whereas the association between TG/HDL mean and CIMT remains robust irrespective of statin use. The inverse association between TG/HDL variability and CIMT observed exclusively among statin-treated patients may reflect treatment-related heterogeneity rather than a direct biological effect of lipid fluctuations on atherosclerotic burden.

**Figure 3:**
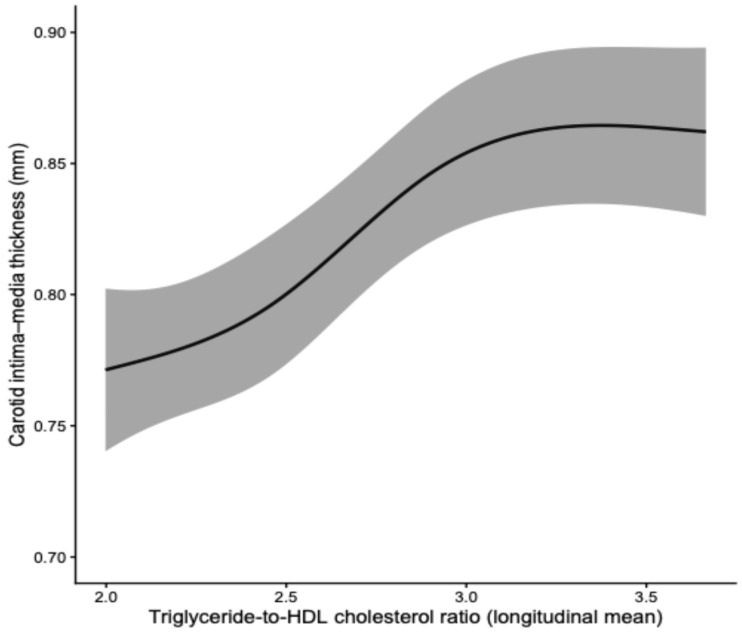
Restricted cubic spline analysis of the association between the longitudinal mean triglyceride-to-HDL cholesterol ratio and carotid intima–media thickness. The solid line represents adjusted predicted values of CIMT derived from the restricted cubic spline model, with the shaded area indicating the 95% confidence interval. The model was adjusted for age, sex, body mass index, diabetes mellitus, hypertension, smoking status, statin therapy, and coronary artery disease.

Restricted cubic spline analysis demonstrated a significant non-linear association between the longitudinal mean TG/HDL ratio and carotid intima–media thickness (*p* for non-linearity = 0.0042), indicating that the relationship between atherogenic dyslipidaemia burden and CIMT is not strictly linear across the TG/HDL spectrum (Figure 3).

## 4. Discussion

In the present study, we examined the associations of both long-term mean levels and visit-to-visit variability of the triglyceride-to-HDL cholesterol (TG/HDL) ratio with carotid intima–media thickness, a structural marker of subclinical atherosclerosis. Among the models tested, higher long-term mean TG/HDL levels were consistently associated with greater CIMT, independent of traditional cardiovascular risk factors, whereas temporal fluctuations in the TG/HDL ratio did not show a consistent association with CIMT. Other lipid and metabolic parameters, including LDL variability and diabetes status, did not demonstrate independent associations in this cohort, and therefore the primary analytical focus was placed on TG/HDL-based metrics, which showed more robust and biologically plausible relationships with CIMT.

These findings suggest that cumulative atherogenic lipid burden, rather than episodic lipid variability, contributes to the development of structural carotid arterial changes over time. This distinction underscores the importance of sustained dyslipidaemia in driving carotid wall thickening beyond the effects of short-term lipid fluctuations.

The relationship between the TG/HDL ratio and CIMT has been previously documented by multiple studies. In pediatric populations, Pacifico et al. reported that higher TG/HDL was associated with increased CIMT and insulin resistance, alongside nonalcoholic fatty liver disease [1]. Shimizu et al. similarly demonstrated associations between TG/HDL and arterial stiffness, particularly in the context of diabetes, in Japanese men [2]. In adults, including individuals with prediabetes, TG/HDL has been proposed as an integrative marker of insulin resistance and atherosclerotic risk, supporting its clinical relevance beyond isolated lipid fractions [3,9]. Collectively, these data corroborate the biological plausibility of TG/HDL as a composite marker linked to adverse vascular remodeling.

Beyond traditional lipid fractions, emerging evidence has highlighted the role of remnant cholesterol and other non-traditional lipid indices in characterizing vascular vulnerability and atherosclerotic burden [10]. Zhao et al. demonstrated that composite lipid parameters were significantly associated with carotid plaque vulnerability and stenosis among patients with acute ischemic stroke, suggesting a link between atherogenic particle burden and advanced plaque phenotypes [11].

Complementing these findings, Di Costanzo et al. reported that higher remnant cholesterol concentrations were associated with increased CIMT in children and adolescents, indicating that atherogenic lipid burden may also influence early arterial wall thickening [12]. Taken together, these observations support the concept that composite lipid markers capture a spectrum of atherosclerotic processes, ranging from diffuse intima–media thickening to plaque-related vascular phenotypes, consistent with the associations observed in the present study.

A major contribution of this study is the analysis of TG/HDL variability measures such as variability independent of the mean (vim), standard deviation (sd), coefficient of variation (cv), and average real variability (arv). In contrast to the consistent association observed for TG/HDL mean, none of the variability measures were found to be independently associated with CIMT, even after adjusting for mean TG/HDL and other traditional cardiovascular risk factors. This may be due to the slowly progressive and chronic nature of CIMT, which probably represents long-term metabolic exposure, as opposed to short-term or visit-to-visit lipids. Prior studies that have examined variability in lipids have often linked variability to acute cardiovascular events or to plaque instability rather than to the thickening of the arterial wall, reinforcing the idea that variability is probably more relevant to the dynamic processes of the vasculature than to its cumulative structural remodeling [13].

In stratified analyses, the association between TG/HDL metrics and carotid intima–media thickness differed according to statin therapy status. Among statin-treated patients, TG/HDL variability showed an inverse association with CIMT, whereas no such association was observed in patients not receiving statin therapy.

This finding should be interpreted with caution and considered exploratory in nature. The observed inverse association may reflect treatment-related heterogeneity, differences in lipid-lowering responses, or confounding by indication, rather than a true protective biological effect of lipid variability. Notably, most prior studies examining the TG/HDL ratio and vascular outcomes have either excluded patients receiving statin therapy or have not specifically evaluated lipid variability, limiting direct comparisons [14,15]. Nevertheless, these observations suggest that the vascular implications of lipid variability may differ in the presence of pharmacological lipid modification, underscoring the importance of considering treatment context when interpreting variability-based lipid metrics. Therefore, this interaction should be regarded as exploratory and hypothesis-generating.

Importantly, the inverse association between TG/HDL variability and CIMT observed exclusively among statin-treated patients should be interpreted as exploratory and hypothesis-generating. Given the observational design, potential confounding by indication, treatment-related heterogeneity, and unmeasured differences in lipid-lowering response cannot be excluded. Therefore, this finding does not imply a protective effect of lipid variability per se but rather suggests that the vascular implications of lipid fluctuations may differ in the presence of pharmacological lipid modification.

The presence of a significant non-linear association between the longitudinal mean TG/HDL ratio and CIMT suggests that the impact of cumulative atherogenic dyslipidaemia on carotid wall thickening may accelerate beyond intermediate TG/HDL levels. The spline analysis indicates a steeper increase in CIMT within the mid-to-upper range of TG/HDL values, followed by a tendency toward plateauing at higher levels, supporting the concept of a threshold-dependent structural response of the arterial wall.

From a pathophysiological perspective, CIMT represents a slowly progressive structural phenotype driven by sustained lipid exposure, smooth muscle cell proliferation, and medial hypertrophy, rather than transient metabolic fluctuations. This provides a biological explanation for why visit-to-visit TG/HDL variability did not independently associate with CIMT, despite its established relevance for more dynamic outcomes such as plaque instability and acute cardiovascular events. Collectively, these findings reinforce the primacy of long-term atherogenic dyslipidaemia burden over short-term lipid variability in relation to CIMT, a marker of chronic vascular remodeling.

Our findings underscored that the long-term mean TG/HDL ratio, an index of cumulative atherogenic dyslipidaemia, is a stronger predictor of subclinical carotid atherosclerosis than short-term lipid fluctuations. Within a longitudinal modeling framework, the current study integrates both level and variability lipid measurements. It clarifies the importance of sustained exposure to alterations in lipid levels rather than to their short-term fluctuations. These findings may aid the development of tools for better assessment of cardiovascular risks and indicate that atherogenic dyslipidaemia may need to be controlled in a sustained manner to prevent structural alterations of the arteries rather than short-term lipid fluctuations, to prevent structural alterations of the arteries.

### Limitations

This study has several limitations that should be acknowledged. First, the retrospective single-center design limits causal inference and may affect generalizability to other populations. Second, carotid intima–media thickness was assessed at a single time point, whereas lipid parameters were measured longitudinally, precluding evaluation of CIMT progression over time. Third, lipid testing frequency and statin exposure, including dose adjustments and adherence, were not fully standardized, and residual confounding from unmeasured lifestyle and clinical factors cannot be excluded.

Nevertheless, the use of repeated lipid measurements over a prolonged follow-up period, the comprehensive assessment of both mean and variability indices, and the application of advanced modeling approaches, including restricted cubic spline analyses, strengthen the robustness of the present findings.

## 5. Conclusions

In this study, the longitudinal mean triglyceride-to-high-density lipoprotein cholesterol (TG/HDL) ratio emerged as the lipid metric most consistently associated with carotid intima–media thickness, underscoring the importance of cumulative atherogenic dyslipidaemia burden in subclinical carotid arterial remodeling. In contrast, visit-to-visit variability of the TG/HDL ratio did not provide incremental information beyond mean TG/HDL levels and established cardiovascular risk factors.

These findings support the concept that CIMT reflects a chronic structural vascular phenotype driven primarily by sustained metabolic exposure rather than short-term lipid fluctuations. The observed context-dependent associations according to statin therapy further highlight that the vascular implications of lipid changes may differ in the presence of pharmacological lipid modification.

Clinically, the TG/HDL ratio represents a simple and readily available marker capable of capturing long-term dyslipidaemia burden and may aid in refining risk stratification for subclinical atherosclerosis. Future prospective studies incorporating serial CIMT assessments and clinical outcomes are warranted to validate these findings and to clarify the role of TG/HDL-based phenotyping in cardiovascular risk prediction.

## Figures and Tables

**Table 1:** Baseline demographic, clinical, and biochemical characteristics of the study population (*n* = 260).

Variable	Overall Population
**Demographic characteristics**	
Age, years	58.4 ± 10.7
Female sex, *n* (%)	121 (46.5)
Body mass index, kg/m^2^	27.9 ± 4.2
**Clinical characteristics**	
Diabetes mellitus, *n* (%)	101 (38.8)
Hypertension, *n* (%)	142 (54.6)
Current smoking, *n* (%)	76 (29.2)
Statin therapy, *n* (%)	109 (41.9)
Coronary artery disease, *n* (%)	84 (32.3)
**Carotid ultrasound**	
CIMT mean, mm	0.95 ± 0.12
**Lipid parameters (longitudinal values)**	
Total cholesterol, mg/dL	201 (176–229) [5.20 (4.55–5.92)]
LDL cholesterol, mg/dL	128 (106–152) [3.31 (2.74–3.93)]
HDL cholesterol, mg/dL	47.8 ± 11.2 [1.24 ± 0.29]
Triglycerides, mg/dL	146 (109–201) [1.65 (1.23–2.27)]
TG/HDL ratio (longitudinal mean)	2.73 (2.38–3.10)
**Lipid variability indices**	
TG/HDL vim	38.1 (29.4–47.8)
TG/HDL sd	0.67 (0.50–0.86)
TG/HDL cv, %	25.2 (19.4–31.6)
TG/HDL arv	0.78 (0.55–1.07)
LDL vim	27.6 (20.4–35.1)
Triglyceride vim	35.9 (25.8–46.7)
HDL vim	17.9 (13.1–22.8)
VLDL vim	33.4 (22.6–45.9)

Data are presented as mean ± standard deviation, median (interquartile range), or number (percentage), as appropriate. Lipid concentrations are reported in mg/dL with corresponding SI units (mmol/L) given in brackets. Abbreviations: CIMT, carotid intima–media thickness; vim, variability independent of the mean; arv, average real variability; cv, coefficient of variation.

**Table 2:** Multivariable linear regression models for the association between TG/HDL mean and CIMT mean.

Variable	Model 1 (Age- and Sex-Adjusted)	Model 2 (Fully Adjusted)
TG/HDL mean	0.051 (0.026 to 0.076) *p* < 0.001	0.044 (0.016 to 0.071) *p* = 0.002
Age (per year)	0.006 (0.005 to 0.007) *p* < 0.001	0.005 (0.004 to 0.006) *p* < 0.001
Female sex	−0.018 (−0.034 to −0.002) *p* = 0.028	−0.014 (−0.029 to 0.001) *p* = 0.071
Body mass index	—	0.003 (0.001 to 0.005) *p* = 0.006
Diabetes mellitus	—	0.021 (0.006 to 0.036) *p* = 0.007
Hypertension	—	0.018 (0.003 to 0.033) *p* = 0.019
Current smoking	—	0.011 (−0.006 to 0.028) *p* = 0.204
Statin therapy	—	−0.009 (−0.024 to 0.006) *p* = 0.236
Coronary artery disease	—	0.027 (0.010 to 0.044) *p* = 0.002
Model R^2^	0.694	0.772
Adjusted R^2^	0.690	0.763

Values are presented as β coefficient (95% confidence interval). Abbreviations: CIMT, carotid intima–media thickness.

**Table 3:** Incremental value of TG/HDL variability indices for CIMT mean beyond TG/HDL mean.

Variable	β Coefficient (95% CI)	*p* Value
TG/HDL mean	0.044 (0.016 to 0.071)	0.002
TG/HDL vim	−0.0005 (−0.0013 to 0.0003)	0.185
Age (per year)	0.005 (0.004 to 0.006)	<0.001
Female sex	−0.014 (−0.029 to 0.001)	0.071
Body mass index	0.003 (0.001 to 0.005)	0.006
Diabetes mellitus	0.021 (0.006 to 0.036)	0.007
Hypertension	0.018 (0.003 to 0.033)	0.019
Current smoking	0.011 (−0.006 to 0.028)	0.204
Statin therapy	−0.009 (−0.024 to 0.006)	0.236
Coronary artery disease	0.027 (0.010 to 0.044)	0.002
Model R^2^	0.772	
Adjusted R^2^	0.763	
ΔR^2^ after adding TG/HDL vim	<0.002	

Abbreviations: CIMT, carotid intima–media thickness; vim, variability independent of the mean.

**Table 4:** Adjusted CIMT mean according to joint categories of TG/HDL mean and TG/HDL vim.

Joint Category (Median Split)	Adjusted CIMT Mean (mm), Estimated Marginal Mean (95% CI)	Pairwise Comparison vs. Reference (Δ, 95% CI)	*p* Value
(1) Low mean / Low vim (reference)	0.9217 (0.8993–0.9441)	—	—
(2) Low mean / High vim	0.9078 (0.8885–0.9272)	−0.0139 (−0.0418 to 0.0140)	0.329
(3) High mean / Low vim	0.9948 (0.9722–1.0173)	+0.0730 (0.0390 to 0.1070)	<0.001
(4) High mean / High vim	0.9669 (0.9433–0.9906)	+0.0452 (0.0102 to 0.0802)	0.011

Covariate-adjusted marginal means were derived from a general linear model adjusted for age, sex, body mass index, diabetes mellitus, hypertension, smoking status, statin therapy, and coronary artery disease. Global test for group effect: *p* < 0.001. Abbreviations: CIMT, carotid intima–media thickness; vim, variability independent of the mean.

**Table 5:** Association of TG/HDL variability with CIMT mean stratified by statin therapy.

Variable	β (95% CI)	*p* Value
**(A) No statin therapy (*n* = 151)**		
TG/HDL mean	0.046 (0.018 to 0.074)	0.001
TG/HDL vim	0.00005 (−0.00085 to 0.00095)	0.918
Model R^2^	0.764	
**(B) Statin therapy (*n* = 109)**		
TG/HDL mean	0.041 (0.009 to 0.073)	0.012
TG/HDL vim	−0.00224 (−0.00375 to −0.00074)	0.003
Model R^2^	0.781	

A formal interaction test between TG/HDL vim and statin therapy was statistically significant (*p* for interaction = 0.011). All models adjusted for age, sex, body mass index, diabetes mellitus, hypertension, smoking status, and coronary artery disease.

## Data Availability

The datasets generated and/or analyzed during the current study are not publicly available due to institutional restrictions on sharing patient-level clinical data, but are available from the corresponding author (A.Y.) on reasonable request and with permission of Karamanoğlu Mehmetbey University Faculty of Medicine.
